# Evolutionary Game Simulation of Knowledge Transfer in Industry-University-Research Cooperative Innovation Network under Different Network Scales

**DOI:** 10.1038/s41598-020-60974-8

**Published:** 2020-03-04

**Authors:** Xia Cao, Chuanyun Li

**Affiliations:** Economics and Management School. Harbin Engineering University, Harbin, China

**Keywords:** Computational science, Computer science, Statistics

## Abstract

This paper takes the industry-university-research cooperation innovation network constructed by the weighted evolutionary BBV model as the research object, which is based on bipartite graph and evolutionary game theory, and constructing the game model of knowledge transfer in the industry-university-research cooperation innovation network, by using the simulation analysis method and analyzing the evolution law of knowledge transfer in the industry-university-research cooperation innovation network under different network scales, three scenarios, the knowledge transfer coefficient and the knowledge reorganization coefficient. The results show that the increase of network scale reduces the speed of knowledge transfer in the network, and the greater the average cooperation intensity of the nodes, the higher the evolution depth of knowledge transfer. Compared with university-research institutes, the evolution depth of knowledge transfer in enterprises is higher, and with the increase of network scale, the gap between the evolution depth of knowledge transfer between them is gradually increasing. Only when reward, punishment and synergistic innovation benefits are higher than the cost of knowledge transfer that can promote the benign evolution of industry-university-research cooperation innovation networks. Only when the knowledge transfer coefficient and the knowledge reorganization coefficient exceed a certain threshold will knowledge transfer behavior emerge in the network. With the increase of the knowledge transfer coefficient and the knowledge reorganization coefficient, the knowledge transfer evolutionary depth of the average cooperation intensity of all kinds of nodes is gradually deepening.

## Introduction

The industry-university-research cooperative innovation network is an important part of the national innovation system. Promoting its stable and orderly development is crucial for optimizing resource allocation, accelerating the transformation of scientific and technological achievements, and enhancing national competitiveness^1^. With the acceleration of the network process, knowledge transfer is gradually transforming from a linear mode to a network mode. Knowledge transfer, as an important way for organizations to acquire necessary knowledge, and establish and maintain competitive advantage, has become particularly prominent in the network^2^. In the process of knowledge transfer in the industry-university-research cooperative innovation network, not only is the cooperative behavior of innovators affected by the network structure^3^ but also the intensity of the cooperative relationship among innovators is closely related^4^, i.e. the connection in the real network has the weight attribute. According to the existing research, a real network with connected weights has both the power-law distribution characteristics of the degree distribution and the power-law distribution characteristics of the strength distribution^5^. The weighted scale-free network constructed based on the weighted evolutionary BBV model can simulate a real network very well^6^. Therefore, taking the weighted scale-free of the industry-university-research cooperative innovation network as the research object, consider the interaction between the cooperative behavior of innovators and network structure, and analyze the evolution law of knowledge transfer in the industry-university-research cooperative innovation network under different network scales and the influence factors of knowledge transfer. This is significant in promoting knowledge flow among innovators in the network and evolution of industry-university-research cooperative innovation networks.

Since Teece first proposed the concept of knowledge transfer^7^, related research on knowledge transfer has attracted wide attention from scholars. For example, the influencing factors of interorganizational knowledge transfer^8,9^, the impact of knowledge transfer on organizational performance^10^, and the methods of promoting interorganizational knowledge transfer^11,12^. In recent years, with the rapid development of network science^13,14^, the related research on knowledge transfer from the perspective of the network has attracted wide attention^15,16^. The researchers mainly focus on the effects of network node attributes^17,18^ and unweighted network structures^19–22^ such as regular network, scale-free network and small world network on knowledge transfer. However, there are still two problems in the study of knowledge transfer in the network, i.e. how knowledge transfers in the network^23,24^, and how the network structure evolves in the process of knowledge transfer^25,26^. Inspired by these studies, a hot topic in the field of complex networks is coevolutionary dynamics, which combines the network structure with the knowledge transfer process of network nodes^27,28^. Many studies have been reported, Cowan *et al*.^29^ and Wang *et al*.^30^ analyzed the influence of knowledge exchange and reciprocity mechanism on knowledge transfer under several established static structure networks based on the knowledge interaction among innovation subjects. Luo *et al*.^31^ and Tur *et al*.^32^ explored the impact of different network structures and feedback processes on knowledge diffusion and network collaboration processes based on the interaction between knowledge diffusion, knowledge creation and network structure.

In addition, with the rapid development of evolutionary game theory^33–37^, the research of evolutionary game from the perspective of complex network is gradually reported^38,39^. After that, some scholars began to explore the problem of knowledge transfer in the network from the perspective of evolutionary game theory. Ozkan-canborat *et al*.^40^ has analyzed the internal mechanism under the interaction of knowledge transfer and participants’ behavior from a macro perspective. Zhang *et al*.^41^ and Mao *et al*.^42^ explored the knowledge transfer mechanism of virtual technology innovation network and school enterprise innovation network respectively from the macro perspective. Zhou *et al*.^43^ and Xu *et al*.^44^ used simulation analysis methods to study the knowledge transfer process mechanism in the cluster innovation network and the R & D team network of manufacturing enterprises from the medium and micro perspective. In these studies, we mainly discuss what factors affect the behavior of innovators and the change of network structure in the process of knowledge diffusion, as well as the knowledge transfer mechanism in the process of network evolution.

Prior efforts on the evolution of knowledge transfer in the network are fruitful. However, the above mentioned studies still have the following shortcomings: (1) The existing research is carried out under the established static network structure, lacking the relevant research under the dynamic changes of network structure. (2) Scholars mostly use weightless networks such as random networks, small-world networks and BA scale-free networks as network models, and lack relevant research under weighted networks with cooperation relationship intensity. (3) Most of the existing studies are based on the macro or meso perspective and regard the innovators in the innovation network as one or several types, ignoring the heterogeneity between innovators, and lacking the micro perspective of the evolution of knowledge transfer in the industry-university-research cooperative innovation network. In view of these, this paper takes the industry-university-research cooperative innovation network constructed by the weighted evolutionary BBV model as the research object, which is based on bipartite graph and evolutionary game theory, and constructing the game model of knowledge transfer in the industry-university-research cooperative innovation network by considering the interaction between knowledge transfer behavior of innovators and the network structure, using the platform of Matlab 2017b for simulation, analyzing the evolution law of knowledge transfer in industry-university-research cooperative innovation network under different network scales, three scenarios, the knowledge transfer coefficient and the knowledge reorganization coefficient. Therefore, this study has important theoretical significance and practical reference for revealing the evolution mechanism of knowledge transfer, promoting knowledge flow among innovators and promoting the evolution of industry-university-research cooperative innovation network.

The remainder of this paper is organized as follows. In Section 2, the game model of knowledge transfer is established in the network. Then, in Section 3, the basic hypothesis of game and the rules of network evolution is proposed. Subsequently, in Section 4, we analyze and discuss the simulation results. Finally, the whole paper is concluded in Section 5.

## Game Model of Knowledge Transfer in Industry-University-Research Cooperative Innovation Network

### Benefit function distribution of knowledge transfer in industry-university-research cooperative innovation network

Based on the research results of Zhou *et al*.^43^ and Xu *et al*.^44^ and the actual situation of the industry-university-research cooperative innovation network, in this paper, factors such as the knowledge reorganization effect, the knowledge synergy effect, the knowledge transfer cost, and the reward and punishment mechanism are incorporated into the knowledge transfer benefit function. For the innovator $i$ and innovator $j$ in the industry-university-research cooperative innovation network, a knowledge transfer benefit function is established.

1 $${\delta }_{i}={\delta }_{i}({S}_{i},{\alpha }_{i}{k}_{j},{\beta }_{i}{S}_{i}{k}_{j},{\gamma }_{i}{k}_{i}^{m}{k}_{j}^{n},{c}_{i}{k}_{i},\lambda {k}_{i},{\theta }_{i})$$

Among them: ${\delta }_{i}$ is the income of the innovators $i$.

${\alpha }_{i}{k}_{j}$ indicates the amount of knowledge acquired by innovator $i$ from innovator $j$, i.e., the direct benefit of knowledge transfer. Among them, ${k}_{j}$ is the amount of knowledge transferred from innovator $j$ to innovator $i$, ${\alpha }_{i}$ is the knowledge transfer coefficient, which depends on the transfer ability, transfer willingness, absorption ability and transfer situation of innovator $j$. That is, knowledge transfer efficiency.

${\beta }_{i}{S}_{i}{k}_{j}$ represents the knowledge reorganization benefits $i$, which means that the innovator $i$ digests, absorbs and re-innovates a small amount of new knowledge from the knowledge acquired from the innovator $j$ on the basis of the understanding of its own knowledge. ${\beta }_{i}$ is the knowledge reorganization coefficient, which reflects the understanding, comprehension and application ability of the knowledge of innovator $i$, and ${S}_{i}$ is the stock of knowledge of innovator $i$.

${\gamma }_{i}{k}_{i}^{m}{k}_{j}^{n}$ represents the knowledge synergistic benefit, referring to the process of communication, cooperation and feedback between innovator $i$ and innovator $j$ to create new knowledge. Among them, ${\gamma }_{i}$ is the coefficient of knowledge synergy, which depends on the cooperation level, innovation ability and knowledge complementarity between innovator $i$ and innovator $j$. $m,n$ is the elasticity coefficient of knowledge transfer between innovator $i$ and innovator $j$, and $m,n > 0$, $m+n=1$.

${c}_{i}{k}_{i}$ indicates the knowledge transfer cost, which refers to the cost and loss of the innovator $i$ in carrying out the knowledge transfer behavior $i$, and ${c}_{i}$ is the knowledge transfer cost coefficient.

$\lambda {k}_{i}$ indicates the reward mechanism, which refers to the reward income obtained by the innovator $i$ in selecting the knowledge transfer, and $\lambda$ is the reward coefficient.

${\theta }_{i}$ indicates the punishment mechanism, which means that the innovator $i$ chooses opportunism, hitchhiking and other punishments when he does not transfer knowledge.

For the convenience of analysis, this paper simplifies the income function as follows:

2 $${\delta }_{i}={\alpha }_{i}{k}_{j}+{\beta }_{i}{S}_{i}{k}_{j}+{\gamma }_{i}{k}_{i}^{m}{k}_{j}^{n}-{c}_{i}{k}_{i}+\lambda {k}_{i}-{\theta }_{i}$$

3 $${\theta }_{i}=\{\begin{array}{c}\begin{array}{cc}0, & {k}_{i} > 0\end{array}\\ \begin{array}{cc}\theta , & {k}_{i}=0\end{array}\end{array}$$

### Game matrix of knowledge transfer in industry-university-research cooperative innovation network

When the game between innovator $i$ and innovator $j$ occurs in the industry-university-research cooperative innovation network, the benefit of each innovator varies under different strategy combinations. On the basis of refs. ^41,44^, this paper establishes a game benefit matrix of knowledge transfer among innovators in the industry-university-research cooperative innovation network (as shown in Table 1).

**Table 1:** Game benefit matrix of knowledge transfer among innovators in industry-university-research cooperation innovation network.

		Innovator $j$	
		Transfer	Non transfer
Innovator $i$	Transfer	${R}_{i}={\alpha }_{i}{k}_{j}+{\beta }_{i}{S}_{i}{k}_{j}+{\gamma }_{i}{k}_{i}^{m}{k}_{j}^{n}-{c}_{i}{k}_{i}+\lambda {k}_{i}$, ${R}_{j}={\alpha }_{j}{k}_{i}+{\beta }_{j}{S}_{j}{k}_{i}+{\gamma }_{j}{k}_{i}^{m}{k}_{j}^{n}-{c}_{j}{k}_{j}+\lambda {k}_{j}$	${P}_{i}=-{c}_{i}{k}_{i}+\lambda {k}_{i}$, ${Q}_{j}={\alpha }_{j}{k}_{i}+{\beta }_{j}{S}_{j}{k}_{i}-\theta$
	Non transfer	${Q}_{i}={\alpha }_{i}{k}_{j}+{\beta }_{i}{S}_{i}{k}_{j}-\theta$, ${P}_{j}=-{c}_{j}{k}_{j}+\lambda {k}_{j}$	${T}_{i}=-\theta$, ${T}_{j}=-\theta$

Strategy 1: When the innovator chooses the (transfer, transfer) strategy, that is, the innovator $i$ chooses the transfer strategy, its income is ${R}_{i}={\alpha }_{i}{k}_{j}+{\beta }_{i}{S}_{i}{k}_{j}+{\gamma }_{i}{k}_{i}^{m}{k}_{j}^{n}-{c}_{i}{k}_{i}+\lambda {k}_{i}$, and the innovator $j$ chooses the transfer strategy, its income is ${R}_{j}={\alpha }_{j}{k}_{i}+{\beta }_{j}{S}_{j}{k}_{i}+{\gamma }_{j}{k}_{i}^{m}{k}_{j}^{n}-{c}_{j}{k}_{j}+\lambda {k}_{j}$.

Strategy 2: When the innovator chooses (transfer, non transfer) strategy, that is, the innovator $i$ chooses the transfer strategy, its income is ${P}_{i}=-\,{c}_{i}{k}_{i}+\lambda {k}_{i}$, and when the innovator $j$ adopts the non transfer strategy, its income is ${Q}_{j}={\alpha }_{j}{k}_{i}+{\beta }_{j}{S}_{j}{k}_{i}-\theta$.

Strategy 3: When the innovator chooses the strategy (non transfer, transfer), the innovator chooses the strategy of non transfer, and its income is ${Q}_{i}={\alpha }_{i}{k}_{j}+{\beta }_{i}{S}_{i}{k}_{j}-\theta$. When the innovator $j$ chooses the strategy of transfer, its income is ${P}_{j}=-\,{c}_{j}{k}_{j}+\lambda {k}_{j}$.

Strategy 4: When the innovator chooses (non transfer, non transfer) strategy, the innovator $i$ and innovator $j$ are affected by opportunism at this time, and their benefits are ${T}_{i}=-\theta$, ${T}_{j}=-\,\theta$, respectively.

## The Basic Hypothesis of Game and the Rules of Network Evolution

### The basic hypothesis of game

On the basis of determining the industry-university-research cooperation innovation network constructed by the empowered evolutionary BBV model, and constructed on the characteristics of the industry-university-research cooperation innovation network and the realistic consideration of the game model, the following assumptions are proposed:

Hypothesis 1: In the industry-university-research cooperation innovation network, enterprises and research institutes are heterogeneous innovators, and the same type of innovation subjects will also have some differences because of their respective scales.

Hypothesis 2: In the industry-university-research cooperation innovation network, the larger the scale of innovators are, the stronger the willingness to transfer knowledge, the stronger the ability to digest and absorb knowledge, the larger the coefficient of knowledge transfer and the coefficient of knowledge reorganization, and the greater the rewards and punishments they receive. At the same time, the larger the scale of innovators are, the more knowledge stock it has and the smaller the cost of the knowledge transfer. In addition, the closer the cooperative relationship between innovators is, the greater the coefficient of knowledge synergy. Compared with enterprises, institutions of higher learning and research as knowledge subjects have higher knowledge transfer, knowledge reorganization, collaborative ability and knowledge stock. Enterprises as economic subjects have a higher cost of knowledge transfer.

Hypothesis 3: In the process of the game, the enterprise or university-research institute $x$ with degree value ${k}_{x}$ only participates in the self-centered and neighborhood-centered game. Moreover, there is a total degree value of ${k}_{x}+1$ in the neighborhood.

Hypothesis 4: In the industry-university-research cooperation innovation network, the innovators are all bounded rational individuals and can only choose transfer (C) and non transfer (D) strategies.

Hypothesis 5: In the industry-university-research cooperation innovation network, all innovators adopt the same strategy to update the rules, and the memory length is 1. That is, the strategy choice of innovators depends on the result of the last game.

### Network evolution rules

Node $m$ in the industry-university-research cooperation innovation network will randomly select a neighbor node $n$ after each round of game for strategy comparison. If $p{r}_{n} > p{r}_{m}$, then node $m$ will imitate the strategy of neighbor node $n$ with probability $W$ in the next round of game. According to the Fermi rule^45^. The imitation probability is as follows:

4 $${W}_{m\to n}=\frac{1}{1+\exp [(p{r}_{n}-p{r}_{m})/K}$$

among them, $K$ represents the intensity of noise, that is, the interference of external factors on the strategy learning process. When $K\to 0$, it means that the external factors will not interfere with the learning strategy of the innovators. In contrast, it means that the innovators can only update their strategies randomly because of the interference of external factors. Considering the effects of the innovator’s income and strategy, this paper chooses the neutral noise factor $K=0.5$ as the simulation parameter value.

When node $m$ chooses the strategy of learning neighbor nodes $n$ by probabilistic $W$, it will disconnect and reconnect with other nodes in the network by probabilistic ϒ_*ms*_. Considering that the innovators are all bounded rational individuals and have certain preferences when choosing partners, this paper uses the reconnection mechanism^46^ with preferential connections to determine the outgoing connection $s$ of node $m$. The stochastic probability is as follows:

5 $${\varUpsilon }_{ms}=\sum _{m\in G}\frac{{{\rm{p}}}_{s}^{\beta }}{{{\rm{p}}}_{m}^{\beta }}$$

among them, ${{\rm{p}}}_{s}$ is the benefit of node $s$, $G$ represents the set where node $m$ is located, $\beta$ indicates preference tendency, and $\beta =0$ indicates no preference connection tendency, that is, random connection. Conversely, the preference is more obvious. This paper chooses high preference $\beta =1$ to simulate.

## Simulation and Analysis of Knowledge Transfer Evolution on Industry-University- Research Cooperative Innovation Network

### Construction of industry-university-research cooperative innovation network

**Figure 1:**
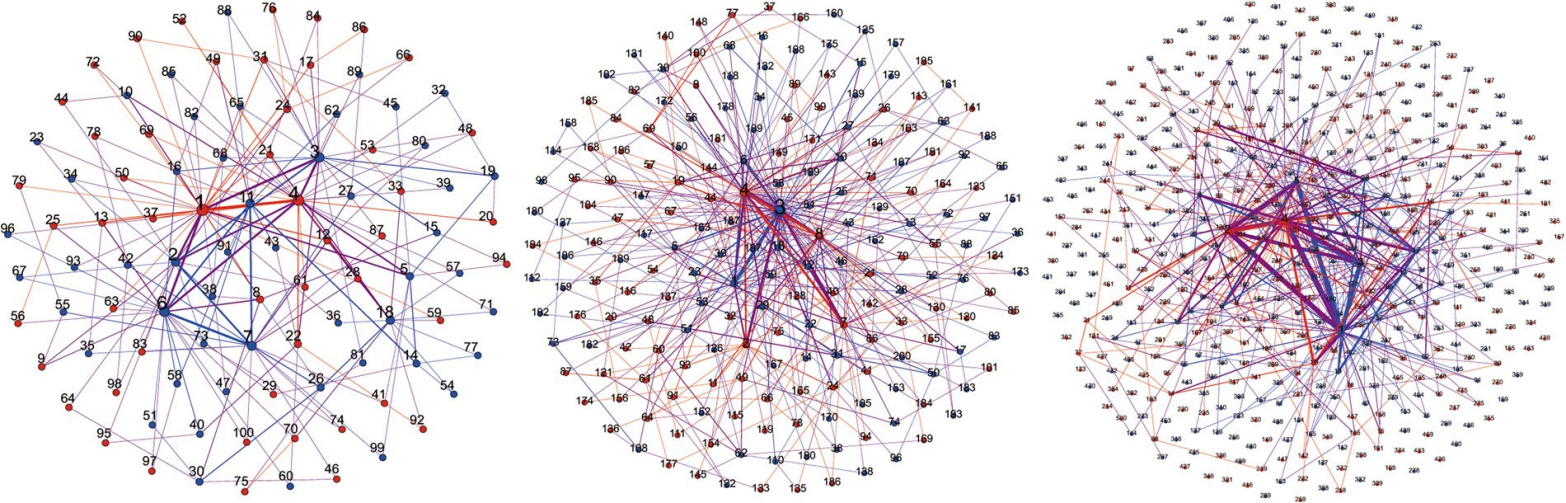
Two-dimensional effect maps of the industry-university-research cooperative innovation network under different network scales.

In this paper, a weighted evolutionary BBV model is used to construct the industry-university-research cooperative innovation network. To reflect the influence of different network scales on knowledge transfer in the industry-university-research cooperative innovation network, this paper sets three network scales: 100, 200 and 500, and describes the process of knowledge transfer in the three network scales by using Matlab and Gephi software. In Fig. 1(a–c), two-dimensional effect maps of the industry-university-research cooperative innovation network under different network scales randomly generated in the early stage of evolution are shown. Among them, the red node represents the enterprise, the blue node represents the university-research institute, the connecting edge represents the direct relationship of the innovators, and the thickness of the connecting edge represents the cooperation relationship intensity between the innovators.

### Simulation steps and parameter settings

1. Simulation steps
  Step 1: Initialize the parameters of evolutionary game and randomly assign the two game strategies of “transfer” and “non transfer” to each node industry-university-research cooperative innovation network. The initial knowledge transfer level is 50%.
  Step 2: In each round of the game, all the innovators play the game with neighbors and accumulate the benefit of each innovators according to the game model.
  Step 3: In each round of the game, all the innovators update their strategies according to the Fermi rule (Eq. 3) and adjust the knowledge transfer objects based on the reconnection mechanism with the preferred connection (Eq. 4). At the end of a round of games, the game strategies and cooperation intensity of each innovator are recorded.
  Step 4: Repeat steps 2 and 3 until the number of Monte Carlo iterations is reached and the simulation is completed.
2. Setting of simulation parameters

To reflect the different characteristics in the knowledge transfer process among the innovators in the industry-university-research cooperative innovation network. In this paper, the enterprise and the university-research institutes are divided into the advantages ($\deg {\rm{ree}} > 4$) and the general ($\deg {\rm{ree}}\le 4$) according to the scale of the innovators. At the same time, combined with the preliminary conclusions of evolutionary game analysis and previous literature^43^, the knowledge transfer coefficient ${\alpha }_{1}$, knowledge recombination coefficient ${\beta }_{1}$, knowledge stock ${S}_{1}$ and knowledge transfer quantity ${k}_{1}$ set at the initial time $(t=0)$ of the advantage university-research institutes obey the uniform distribution of $(0.35,0.45)$, $(0.02,0.03)$, $(14,15)$ and $(9,11)$ respectively. The knowledge transfer coefficient ${\alpha }_{2}$, knowledge recombination coefficient ${\beta }_{2}$, knowledge stock ${S}_{2}$ and knowledge transfer quantity ${k}_{2}$ at the initial time $(t=0)$ of advantage enterprise obey the uniform distribution of $(0.30,0.40)$, $(0.015,0.025)$, $(13,14)$ and $(8,10)$ respectively. The knowledge transfer coefficient ${\alpha }_{1}$, knowledge reorganization coefficient ${\beta }_{1}$, knowledge stock ${S}_{1}$ and knowledge transfer quantity ${k}_{1}$ at the initial time $(t=0)$ of general university-research institutes obey the uniform distribution of $(0.25,0.35)$, $(0.010,0.020)$, $(12,13)$ and $(7,9)$ respectively. The knowledge transfer coefficient ${\alpha }_{2}$, knowledge recombination coefficient ${\beta }_{2}$, knowledge stock ${S}_{2}$ and knowledge transfer quantity ${k}_{2}$ at the initial time $(t=0)$ of general enterprise obey the uniform distribution of $(0.20,0.30)$, $(0.005,0.015)$, $(11,12)$ and $(6,8)$ respectively. In addition, the reward coefficient $\lambda$, elasticity coefficient $m,n$ and penalty intensity $\theta$ of each innovator are set to be the same, that is, ${\lambda }_{1}={\lambda }_{2}=\lambda$, ${m}_{1}={m}_{2}=m$, ${n}_{1}={n}_{2}=n$ 和${\theta }_{1}={\theta }_{2}=\theta$. Among them, the reward coefficient $\lambda$ and elasticity coefficient $m,n$ are 0.1 and 0.5, respectively, and the penalty intensity $\theta$ obeys the uniform distribution of $(0,2)$. Referring to the literature 44, considering three evolutionary game scenarios of knowledge transfer, the knowledge synergy coefficients and knowledge transfer costs of advantage university-research institutes, advantage enterprises, general advantage university-research institutes and general enterprises are set up as shown in Table 2.

**Table 2:** Setting of simulation parameters of knowledge transfer evolutionary game in industry- university-research cooperative innovation network.

Parameter	Advantage university- research institutes ${c}_{1}$	Advantage enterprise ${c}_{2}$	General advantage university-research institutes ${c}_{1}$	General enterprise ${c}_{2}$	Advantage university-research institutes ${\gamma }_{1}$	Advantage enterprise ${\gamma }_{1}$	General advantage university- research institutes ${\gamma }_{2}$	General enterprise ${\gamma }_{2}$
D1	[0.10,0.20]	[0.15,0.25]	[0.20,0.30]	[0.25,0.35]	[0.15,0.25]	[0.10,0.20]	[0.05,0.15]	[0.00,0.10]
D2	[0.40,0.50]	[0.45,0.55]	[0.50,0.60]	[0.55,0.65]	[0.30,0.40]	[0.25,0.35]	[0.20,0.30]	[0.15,0.25]
D3	[0.45,0.55]	[0.50,0.60]	[0.55,0.65]	[0.60,0.70]	[0.15,0.25]	[0.10,0.20]	[0.05,0.15]	[0.00,0.10]

### Simulation results and discussion

#### The influence of the three scenarios on the knowledge transfer evolution of the industry-university-research cooperation innovation network under different network scales

**Figure 2:**
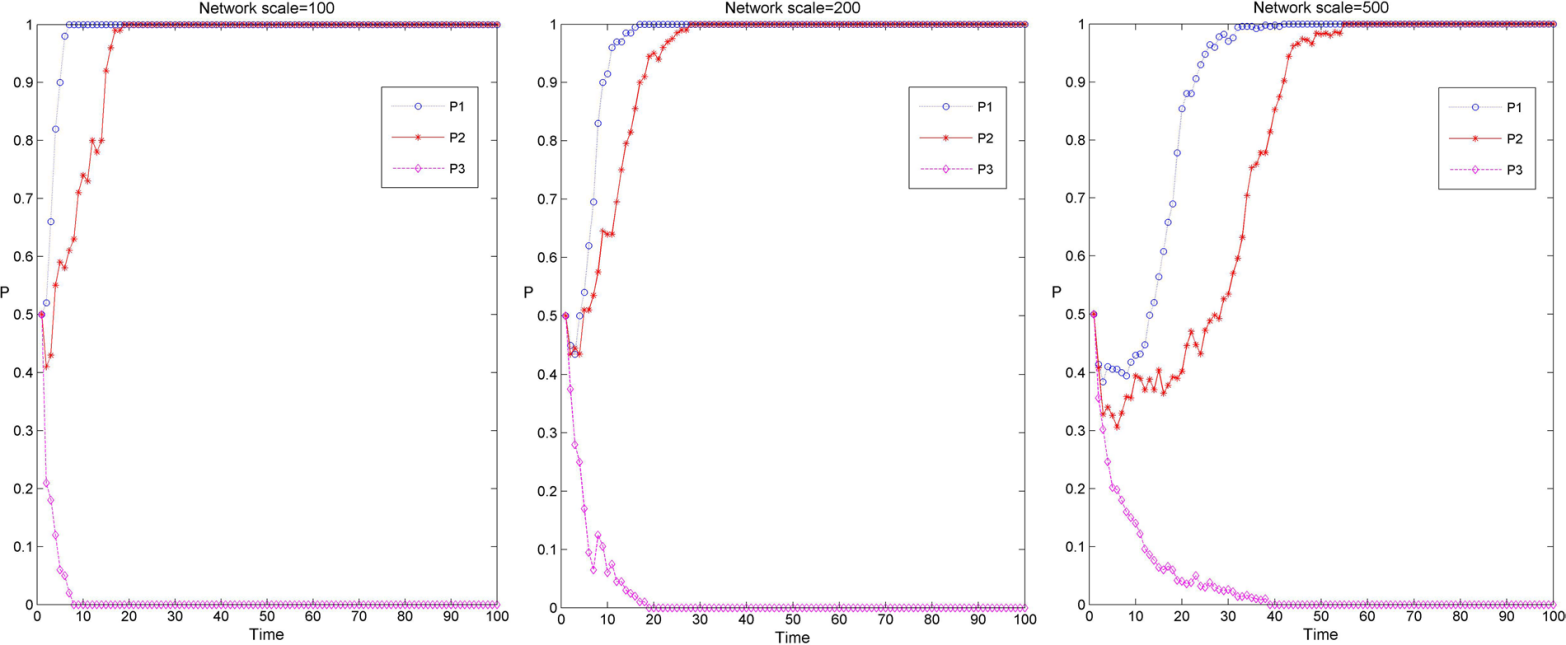
Evolution simulation results of knowledge transfer in the industry-university-research cooperation innovation network under different network scales and three scenarios.

Figure 2 shows the simulation results of the knowledge transfer evolution of the industry-university-research cooperation innovation network under different network scales and three scenarios. Among them, P1, P2 and P3 correspond to the simulation evolution curve under the three parameter settings of D1, D2 and D3 in Table 2. When the sum of rewards and penalties of the innovators’ knowledge transfer strategy is less than the cost of knowledge transfer. That is, the cost of knowledge transfer behavior of the enterprises and the university-research institutes cannot be compensated under the network environment, so that the result of network evolution converges to 0 (as shown in the evolutionary curve P3 in Fig. 2). Enterprise and university research institutes in the network choose the non transfer strategy, which means that there is no knowledge interaction among innovators. When the reward and punishment of innovators’ knowledge transfer strategy and the level of collaborative innovation are relatively high, the sum of reward and punishment of innovators’ knowledge transfer strategy and the benefit of collaborative innovation is greater than the cost of knowledge transfer. The industry-university-research cooperation innovation network can rapidly evolve to a 100% steady state in the process of knowledge transfer (as shown in the evolution curve P1 in Fig. 2). When the rewards and penalties of the innovators’ knowledge transfer strategy are less than the cost of knowledge transfer and the sum of synergistic benefits and the knowledge is larger than the cost of knowledge transfer, the industry-university-research cooperation innovation network can evolve to a 100% steady state at a relatively slow speed in the process of knowledge transfer (as shown in the evolution curve P2 in Fig. 2). In this case, although the reward and punishment under the network environment is weak, the synergistic innovation ability among innovators is strong, which can make up for the cost of knowledge transfer and produce benefits.

In addition, it can be found from Fig. 2 that the evolution simulation of knowledge transfer in the industry-university-research cooperation innovation network has similar evolution results under different network scales. In the same situation, along with the increase of network scale, the time of knowledge transfer evolving to a stable stage in the industry-university-research cooperation innovation network is gradually longer, and the evolution speed of knowledge transfer is gradually slower. This may be because the node degree, average weighted degree and shortest path in small-scale networks are relatively small, and the efficiency of information transmission is high. In large-scale networks, the more cooperative relationships there are among the nodes, the more uneven distribution and heterogeneity of the nodes, the more complex the process of revenue comparison and strategy learning among the nodes in the game process, the lower the efficiency of information transmission and the slower the speed of knowledge transfer^44^.

**Figure 3:**
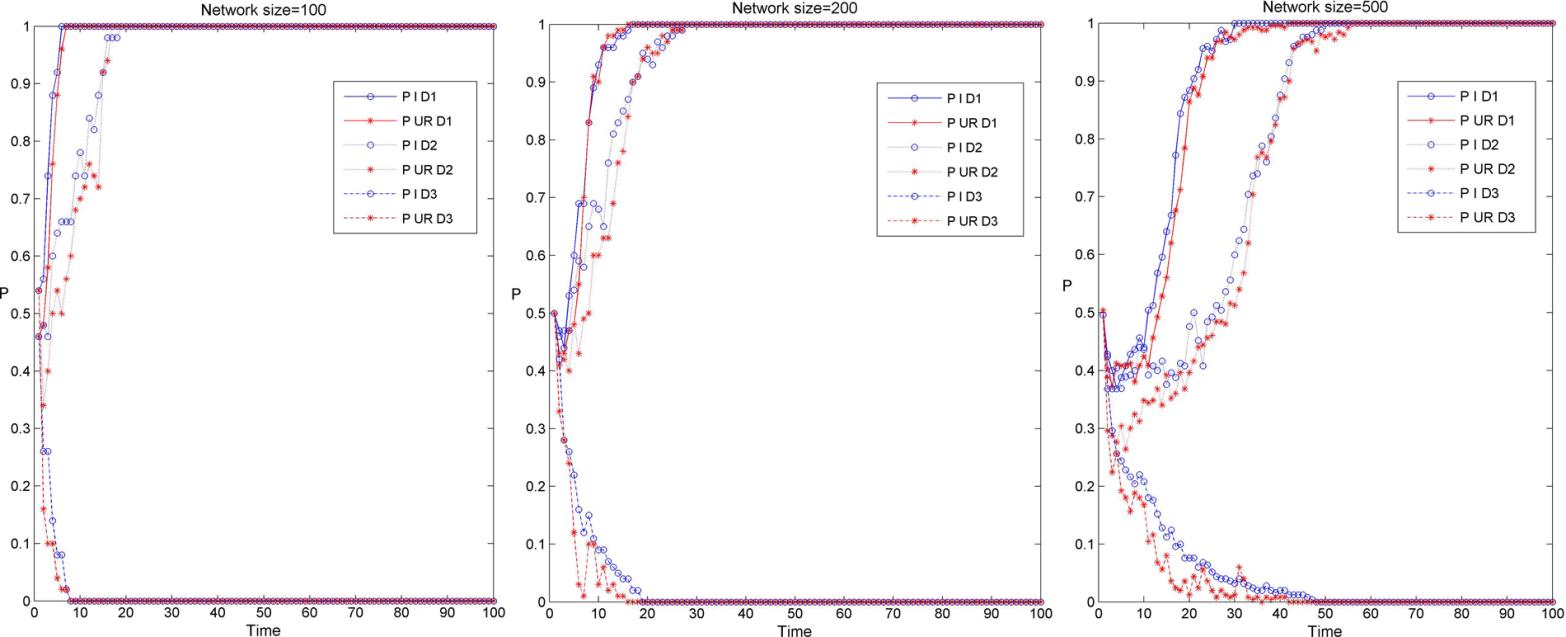
Evolution simulation results of knowledge transfer between enterprises and university-research institutes in industry-university-research cooperation innovation network under different network scales and three scenarios.

Figure 3 shows the simulation results of the knowledge transfer evolution of enterprises and university-research institutes in the industry-university-research cooperation innovation network under different network scales and three scenarios. Among them, Fig. 3(a–c) shows the simulation results of knowledge transfer evolution in three network scales, and the red and blue curves respectively show the evolution trend of knowledge transfer of university-research institutes and enterprises under three scenarios. From Fig. 3, it can be found that when the evolution depth tends to 0, before the evolution to stability, the evolution depth of enterprise knowledge transfer is relatively deep, and the evolution time to stability is relatively long under the same evolution time. In addition, with the increase of the network scale, the time for the knowledge transfer of enterprises and university-research institutes to evolve to stability has gradually become longer, and the degree of differentiation of the evolution depth of knowledge transfer between them has gradually increased. This may be that in small-scale networks, the degree of enterprise is relatively small, and there are fewer subjects for knowledge interaction. At the same time, the amount of knowledge transfer in university-research institutes is relatively large. Enterprises can rapidly improve their knowledge level by cooperating with university-research institutes, thus having a higher preference for knowledge transfer, which makes the evolution depth of knowledge transfer in enterprises higher. In large-scale networks, there are more innovators for knowledge interaction in enterprises, while the attraction of enterprises brought by the knowledge level of institutions of university-research institutes is gradually declining, and the preference for knowledge transfer is also gradually weakening, so that the gap between enterprises and university-research institutes in the evolution depth of knowledge transfer is gradually increasing.

**Figure 4:**
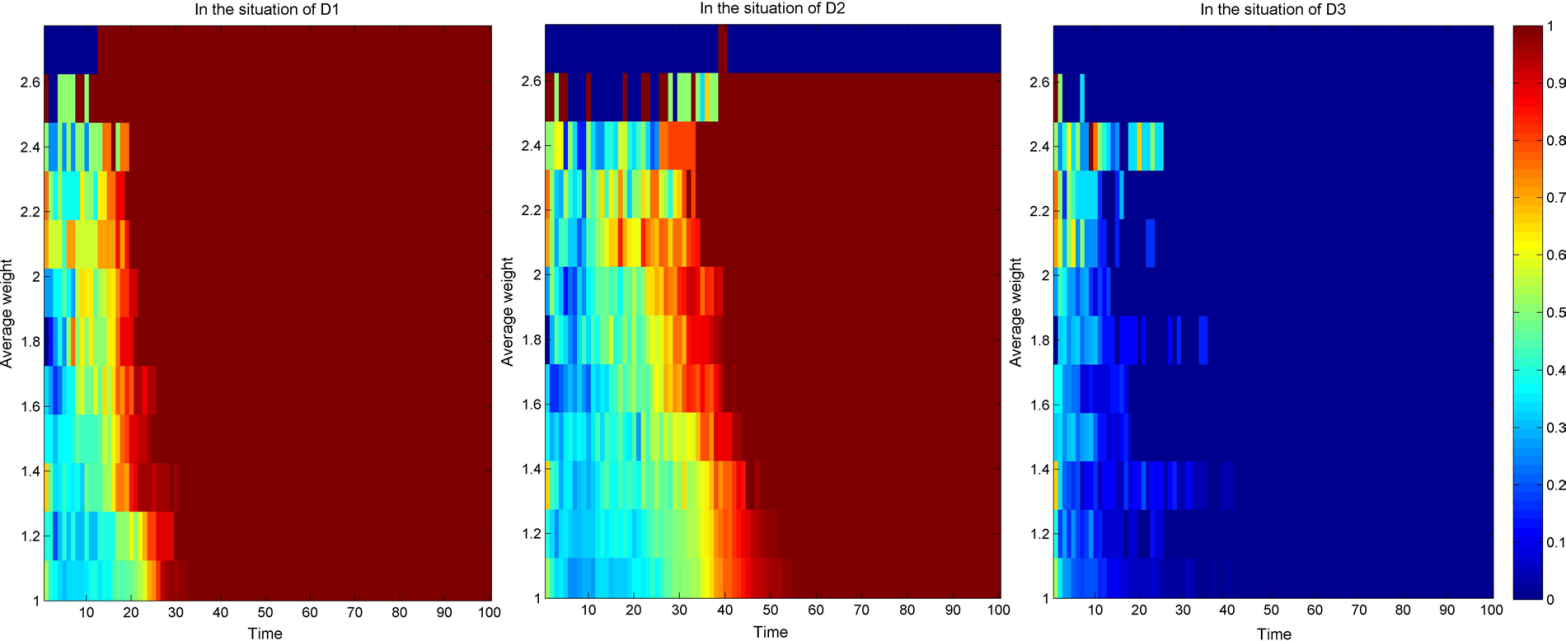
Thermogram of the knowledge transfer evolution simulation results of the average cooperation intensity of the nodes in the industry-university-research cooperation innovation network under three scenarios.

Due to different network scales, the knowledge transfer evolution of the average cooperation intensity of the nodes in the industry-university-research cooperation innovation network has similar simulation results. To intuitively know the evolution of knowledge transfer with average cooperation intensity, this paper selects when the network scale is 500. As shown in Fig. 4(a–c), a thermogram of the knowledge transfer evolution simulation results of the average cooperation relationship intensity of the nodes in the industry-university-research cooperation innovation network under three scenarios is presented when the network scale is 500. Among them, the abscissa represents the number of network evolution, the ordinate represents the average cooperation intensity of the nodes, and the color in the graph represents the evolution depth of knowledge transfer. From Fig. 4, it can be found that before evolution to stability, under the same evolutionary time, the greater the average cooperation intensity of the nodes, the deeper the evolution depth of the knowledge transfer. This is because the greater the average cooperation intensity of the nodes in the network, the more stable the cooperation relationship between the neighbors directly connected to them, and the deeper the trust degree of knowledge acquisition through the cooperation relationship between them^47^, which makes the innovators more inclined to choose the knowledge transfer strategy. Therefore, the node with the greater average cooperation intensity is more favorable to promote the knowledge transfer evolution in the industry-university-research cooperation innovation network.

#### The influence of the knowledge transfer coefficient on the knowledge transfer evolution of the industry-university-research cooperation innovation network under different network scales

**Figure 5:**
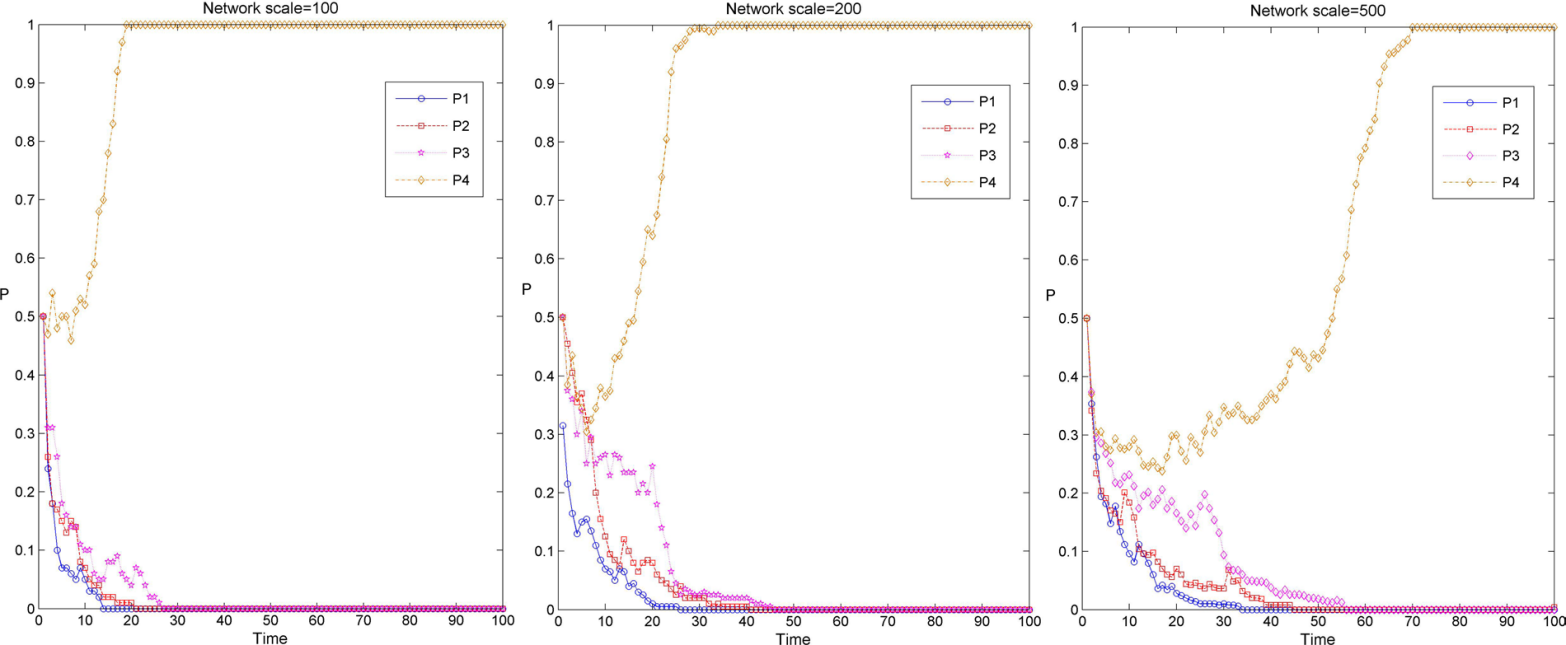
Evolution simulation results of knowledge transfer in the industry-university-research cooperation innovation network under different network scales and knowledge transfer coefficients.

On the basis of the situation in which the evolutionary depth of knowledge transfer in the industry-university-research cooperation innovation network is 0, the knowledge transfer coefficients $\alpha$ of advantage university-research institutes, advantage enterprise, general university-research institutes, and general enterprise obey the uniform distribution of $(0.05+i,0.15+i)$, $(0.10+i,0.20+i)$, $(0.15+i,0.25+i)$ and $(0.20+i,0.30+i)$ in turn. The values of $i$ are 0.0, 0.2, 0.4 and 0.6, respectively, to adjust the knowledge transfer coefficient $\alpha$ of all kinds of innovators, expressed by P1, P2, P3 and P4, respectively. The simulation results of the knowledge transfer evolution in the industry-university-research cooperation innovation network under different network scales and knowledge transfer coefficients are obtained, as shown in Fig. 5. Among them, Fig. 5(a–c) respectively shows the simulation results of knowledge transfer evolution under three network scales, P1, P2, P3 and P4 represent the simulation evolution curves under four knowledge transfer coefficients.

From Fig. 5, it can be found that when the final results of network evolution converge to 0 under the same network scale, with the increase of knowledge transfer coefficient, the time for the evolution of the industry-university-research cooperation innovation network to be stable gradually becomes longer. This is because the increase of the knowledge transfer coefficient enhances the knowledge transfer ability, the knowledge transfer willingness and the knowledge absorption ability of the innovators, and the innovators’ knowledge transfer behavior preference gradually strengthens, so that the innovators’ cooperative behavior preference changes from a “non transfer” dominant direction to a “transfer” dominant direction. Under the same knowledge transfer coefficient, along with the increase of the network scale, the time for the evolution of the industry-university-research cooperation innovation network to stability gradually becomes longer. This is due to the increase of the network scale, the average path between innovators increases gradually, and the efficiency of information transmission decreases gradually. In the process of network games, innovators are faced with more complex revenue comparison and strategy selection, which leads to the slow evolution of knowledge transfer and the time from the evolution of the industry-university-research cooperation innovation network to stabilization gradually becomes longer. In addition, it can be found that only when $i$ is 0.6, that is, when the knowledge transfer coefficient is the maximum, the evolution result of knowledge transfer in the industry-university-research cooperation innovation network converges to 1. It shows that only when the knowledge transfer coefficient is higher than a certain threshold will the knowledge transfer behavior of innovators emerge in the network^48^.

**Figure 6:**
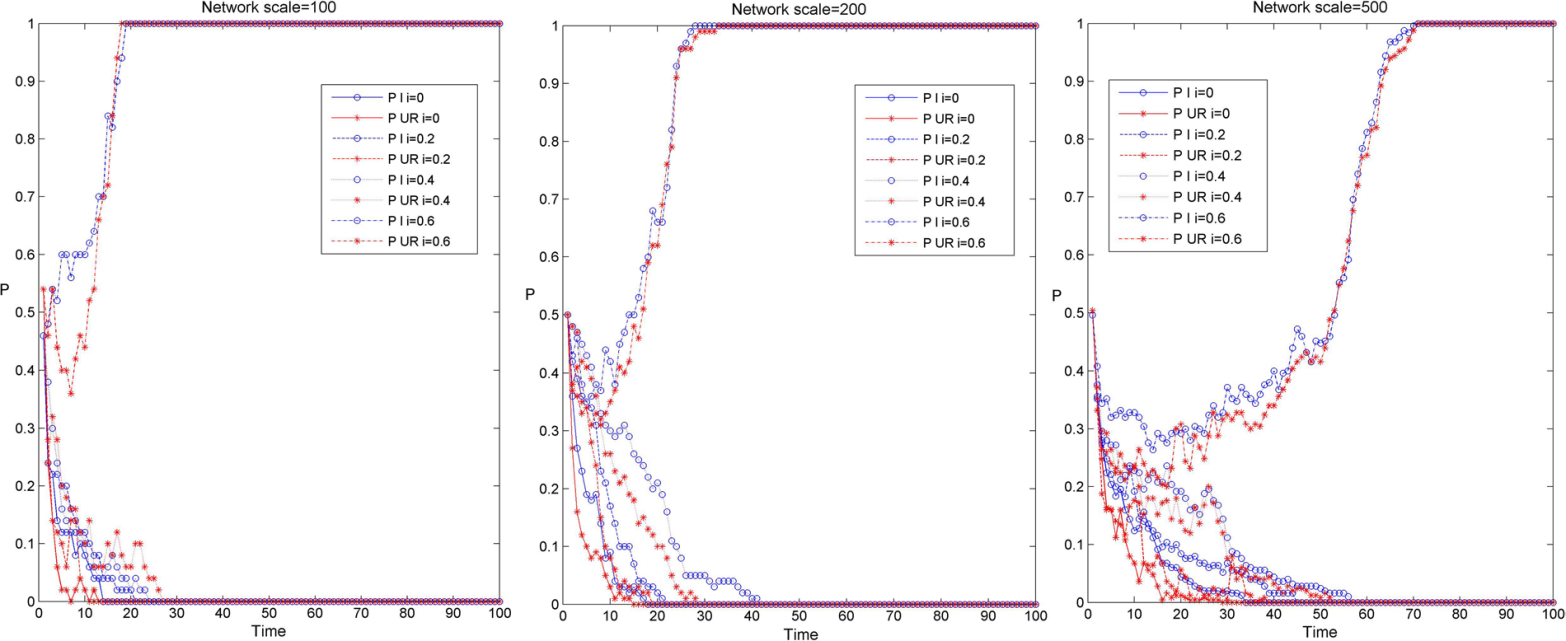
Evolution simulation results of knowledge transfer between university-research institutes in the industry-university-research cooperation innovation network under different network scales and knowledge transfer coefficients.

Figure 6 shows the simulation results of the knowledge transfer evolution of enterprises and university-research institutes in the industry-university-research cooperation innovation network under different network scales and knowledge reorganization coefficients. Among them, Fig. 6(a–c) shows the simulation results of knowledge transfer evolution under three network scales, and the red and blue curves respectively shows the evolution trend of knowledge transfer of university-research institutes and enterprises under four knowledge transfer coefficients. From Fig. 6, it can be found that before the evolution to stability, when the evolution results converge to 1, the depth of knowledge transfer evolution of enterprises and university-research institutes is almost the same. When the evolutionary depth tends to 0, the depth of knowledge transfer evolution in enterprises is relatively higher than that in university-research institutes, and with the increase of network scale, the gap of knowledge transfer evolution depth between enterprises and university-research institutes is also increasing. This is because in the process of knowledge transfer, enterprises in the industry-university-research cooperation innovation network have a better thirst for knowledge and are more willing to acquire more heterogeneous knowledge through knowledge transfer, which makes enterprises have a higher preference for knowledge transfer behavior and is more critical to maintain the stable development of knowledge transfer in the network. In addition, under the same knowledge transfer coefficient, with the increase of network scale, the time for knowledge transfer of enterprises and university-research institutes to evolve to a stable state is gradually increasing. This is because with the increase of network scale, the degree value of enterprises and university-research institutes is increasing, the average path length of network is getting longer, and the efficiency of information transmission is decreasing, so that the time of knowledge transfer evolution is getting longer.

**Figure 7:**
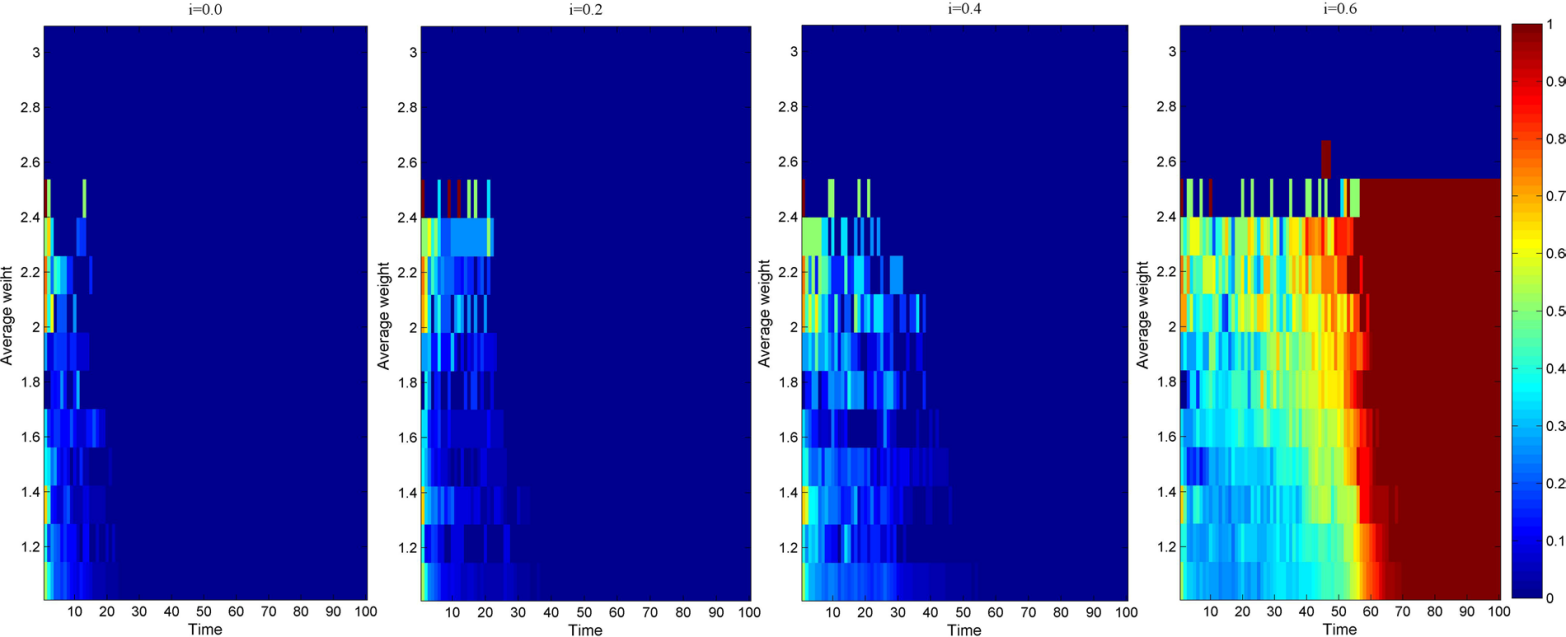
Thermogram of knowledge transfer evolution simulation results of the average cooperation intensity of the nodes in the industry-university-research cooperation innovation network under different knowledge transfer coefficients.

Figure 7(a–d) respectively shows the simulation results of the knowledge transfer evolution of the average cooperation intensity of nodes in the industry-university-research cooperation innovation network under different knowledge transfer coefficients when the network scale is 500. From Fig. 7, it can be found that before the evolution to stability, when the knowledge transfer coefficient is the same, the greater the average cooperation intensity of the nodes, the deeper the evolution depth of the knowledge transfer. This is because the greater the average cooperation intensity of the nodes in the network, the more solid the cooperation relationship between the directly connected neighbors, and the more the partners are inclined to choose the knowledge transfer strategy to enhance their knowledge level. Before the evolution to stability, when the evolution time is the same, with the increase of the knowledge transfer coefficient, the knowledge evolution depth under the intensity of various average cooperative relationships is gradually increasing. This is due to the improvement of knowledge transfer ability and willingness to transfer knowledge, and the preference of knowledge transfer behavior of each type of average cooperation intensity node is gradually increasing so that the intensity of the cooperation relationship is gradually increasing.

#### The influence of the knowledge reorganization coefficient on the evolution of knoNwledge transfer in the industry-university-research cooperation innovation network under different network scales

**Figure 8:**
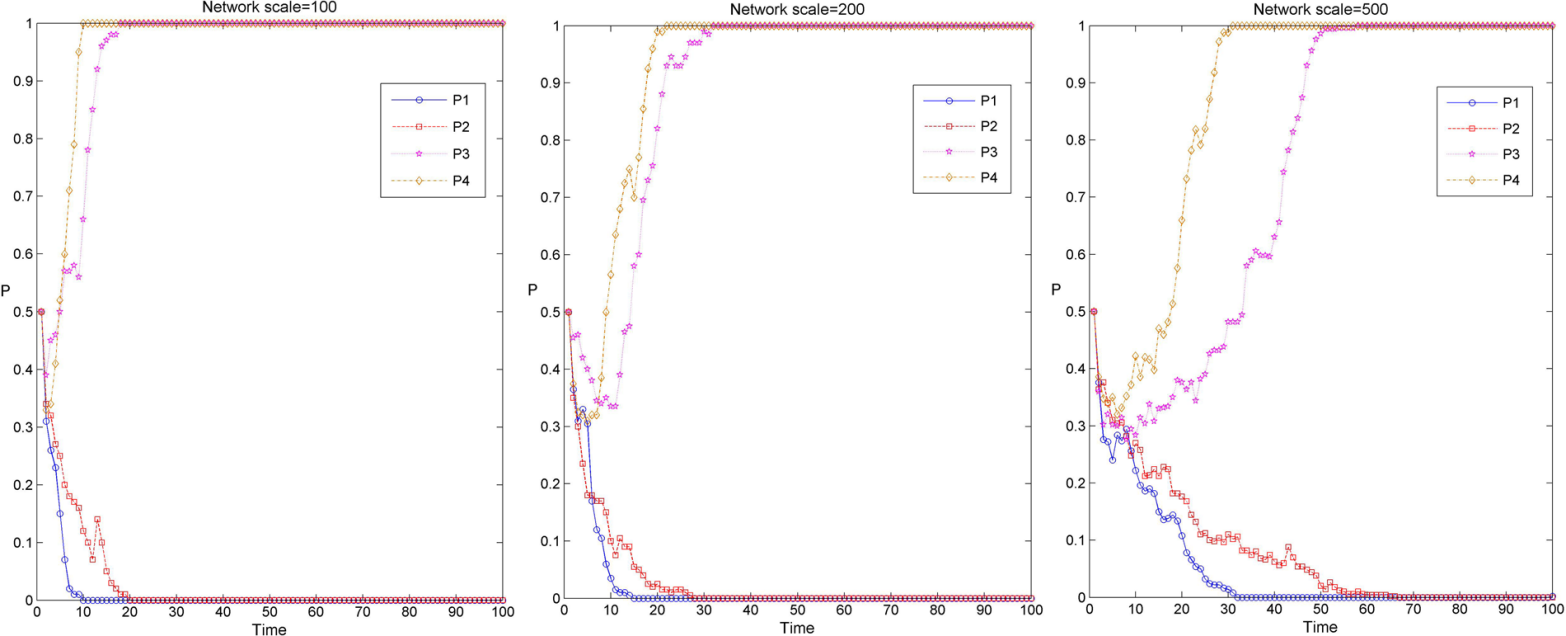
Evolution simulation results of knowledge transfer in the industry-university-research cooperation innovation network under different network scales and knowledge reorganization coefficients.

On the basis of the situation in which the evolutionary depth of knowledge transfer in the industry-university-research cooperation innovation network is 0, the knowledge reorganization coefficients $\beta$ of advantage university-research institutes, advantage enterprise, general university-research institutes, general enterprise obey the uniform distribution of $(0.02+j,0.03+j)$, $(0.015+j,0.025+j)$, $(0.01+j,0.02+j)$ and $(0.005+j,0.015+j)$ in turn. The values of $j$ are 0.01, 0.02, 0.03, and 0.04, respectively, to adjust the knowledge reorganization coefficients $\beta$ of all kinds of innovators, expressed by P1, P2, P3 and P4, respectively. The simulation results of the knowledge transfer evolution in the industry-university-research cooperation innovation network under different network scales and knowledge reorganization coefficients are obtained, as shown in Fig. 8. Among them, Fig. 8(a–c) respectively shows the evolution results of knowledge transfer under three network scales. P1, P2, P3 and P4 represent the simulation evolution curves under four knowledge reorganization coefficients.

From Fig. 8, it can be found that when the final results of network evolution converge to 0 under the same network scale, with the increase of the knowledge reorganization coefficient, the time of knowledge transfer evolving to stability is gradually longer. However, when the final results of network evolution converge to 1, with the increase of the knowledge reorganization coefficient, the time of knowledge transfer evolving to stability is gradually shortened. This may be due to the increase of the knowledge reorganization coefficient, which makes the ability of innovators to understand, comprehend and apply knowledge gradually enhanced. The new knowledge acquired through digestion, absorption and reinnovation increases gradually, so that the knowledge transfer behavior preference of the innovator is gradually enhanced and then promotes the steady development of knowledge transfer in the industry-university-research cooperation innovation network. With the same knowledge reorganization coefficient, along with the increase of network scale, the time of knowledge transfer evolving to stability in the industry-university-research cooperation innovation network gradually becomes longer. This is because with the increase of the network scale, the level of knowledge application ability of innovators is not uniform, and the average path length between innovators is gradually increasing, which affects the transmission efficient knowledge information in the network, thus reducing the evolution speed of knowledge transfer and delaying the evolution time of knowledge transfer. In addition, it can be found from the graph that the final result of knowledge transfer evolution of the industry-university-research cooperation innovation network converges to 1 only when $j$ is 0.03. This also shows that only when the knowledge reorganization coefficient is higher than a certain threshold, the innovator has a certain ability of understanding, comprehending and applying the knowledge, so that the knowledge transfer behavior will emerge in the network.

**Figure 9:**
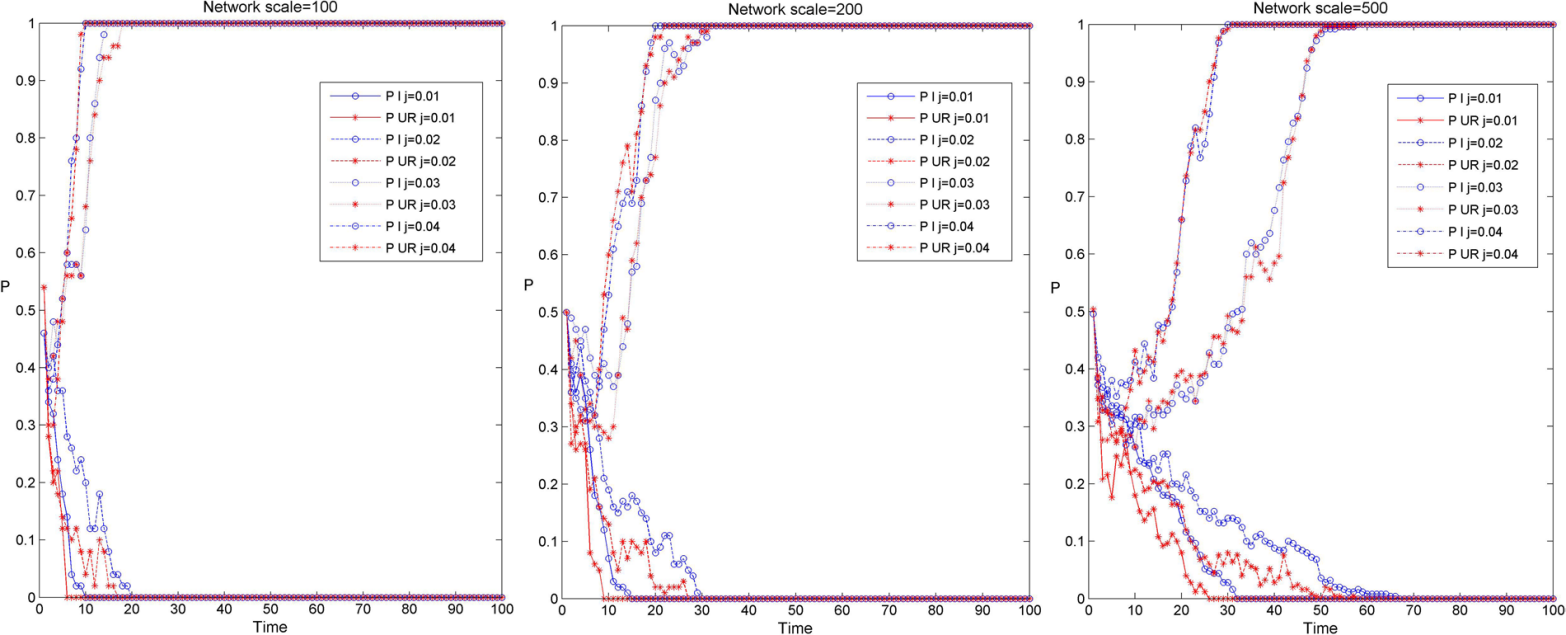
Evolution simulation results of knowledge transfer of enterprises and university- research institutes in the industry-university-research cooperation innovation network under different network scales and knowledge reorganization coefficients.

Figure 9 shows the simulation results of the knowledge transfer evolution of enterprises and university-research institutes in the industry-university-research cooperation innovation network under different network scales and knowledge reorganization coefficients. Among them, Fig. 9(a–c) respectively shows the evolution results of knowledge transfer under three network scales, and the red and blue curves respectively shows the evolution trend of knowledge transfer of university-research institutes and enterprises under four knowledge reorganization coefficients. From Fig. 9, it can be found that before the network evolves to stability, when the evolution results converge to 1, the depth of knowledge evolution of enterprises and university-research institutes is almost the same. When the evolution results converge to 0, the knowledge evolution depth of enterprises is deeper than that of enterprises and university research institutes, and the larger the network scale, the greater the gap between the two. This is because in the process of knowledge transfer evolution in the industry-university-research cooperation innovation network, enterprises prefer to acquire more new knowledge through the process of knowledge reorganization to promote the rapid development of enterprises themselves. Therefore, enterprises have a higher preference for knowledge transfer behavior, which is very important to maintain the evolution of knowledge transfer in the network. In addition, with the same knowledge reorganization coefficient, along with the increase of network scale, the time for knowledge transfer of enterprises and university-research institutes to evolve to a stable state gradually becomes longer. This is due to the increase of network scale, the gap enterprises and university-research institutes on knowledge understanding, comprehension and application ability is gradually increasing, and the number of partners of some enterprises and university-research institutes is also increasing, so that the process of network game becomes more complex, affecting the decision-making of enterprises and university-research institutes, then reduces the speed of knowledge transfer evolution and prolongs the time of knowledge transfer evolution.

**Figure 10:**
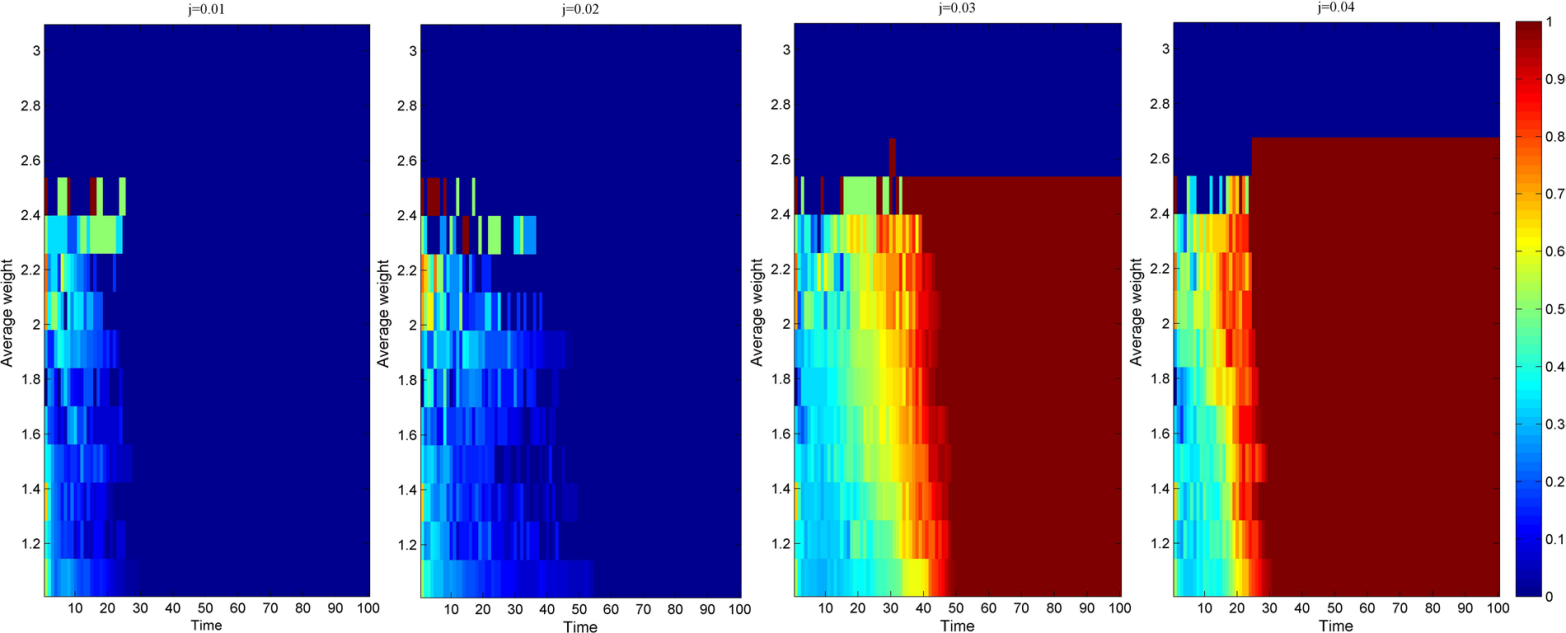
Evolution simulation results of knowledge transfer in the industry-university-research cooperation innovation network under different knowledge reorganization coefficients.

Figure 10(a–d) respectively shows the simulation results of the knowledge transfer evolution of the average cooperation intensity of nodes in the industry-university-research cooperation innovation network under different knowledge reorganization coefficients when the network scale is 500. From Fig. 10, it can be found that before the evolution to stability, when the knowledge reorganization coefficient is the same, the greater the average cooperation intensity of the nodes, and the deeper the evolution depth of the knowledge transfer. This may be that the greater the average cooperation intensity of the nodes in the network, the higher the degree of mutual understanding^49^, and the more likely it is to acquire more new knowledge through the process of knowledge reorganization, which makes the partners more inclined to choose knowledge transfer strategies to promote their knowledge level. In addition, before the evolution to stability, when the evolution time is the same, along with the increase of the knowledge reorganization coefficient, the depth of knowledge evolution under the average cooperation intensity is gradually deepening. This is because with the improvement of knowledge understanding, comprehension and application ability of innovators, all kinds of average cooperation intensity nodes tend to choose knowledge transfer strategies to improve their knowledge level so that the depth of knowledge evolution under the average cooperation intensity is gradually deepening.

## Conclusions

This paper takes the industry-university-research cooperation innovation network constructed by the weighted evolution BBV model as the research object. Based on the bipartite graph and the evolutionary game theory, and constructing the game model of knowledge transfer in the industry-university-research cooperation innovation network, by using simulation analysis method, studies the evolution process of knowledge transfer in the industry-university-research cooperation innovation network under different network scales, three scenarios, the knowledge transfer coefficient and the knowledge reorganization coefficient, and the following conclusions are drawn:

1. Under the influence of different network scales, three scenarios, the knowledge transfer coefficient and the knowledge reorganization coefficient, along with the increase of the network scale, the slower the speed of knowledge transfer in the network is, the longer the time of knowledge transfer evolving to stability. At the same evolutionary time, the greater the average cooperation intensity of the nodes are, the deeper the evolutionary depth of the knowledge transfer. When the evolutionary time is the same, the evolutionary depth of knowledge transfer in enterprises is higher than that in university-research institutes. Meanwhile, with the increase of the network scale, the gap in the depth of knowledge transfer between the two has gradually increased.
2. With the relationship between reward and punishment, synergistic innovation income and knowledge transfer cost together determine the result of the evolution of knowledge transfer in the industry-university-research cooperation innovation network. Only when the reward, punishment and synergistic innovation benefits are higher than the knowledge transfer cost can the benign evolution of the industry-university-research cooperation innovation network be promoted.
3. Only when the knowledge transfer coefficient and the knowledge reorganization coefficient exceed a certain threshold will knowledge transfer behavior emerge in the industry-university-research cooperation innovation network. At the same time, with the increase of the knowledge transfer coefficient and the knowledge reorganization coefficient, the depth of knowledge transfer evolution under various types of average cooperation intensity is gradually deepening.
